# Anticoagulant and Antithrombotic Properties of Three Structurally Correlated Sea Urchin Sulfated Glycans and Their Low-Molecular-Weight Derivatives

**DOI:** 10.3390/md16090304

**Published:** 2018-08-30

**Authors:** Ariana A. Vasconcelos, Isabela D. Sucupira, Alessandra L. Guedes, Ismael N. Queiroz, Flavia S. Frattani, Roberto J. Fonseca, Vitor H. Pomin

**Affiliations:** 1 Program of Glycobiology, Institute of Medical Biochemistry Leopoldo de Meis, Federal University of Rio de Janeiro, Rio de Janeiro 21941-590, RJ, Brazil; arianaavasconcelos@gmail.com (A.A.V.); isabela.sucupira@gmail.com (I.D.S.); alessandra.guedes@bioqmed.ufrj.br (A.L.G.); mael.nilo@hotmail.com (I.N.Q.); 2 University Hospital Clementino Fraga Filho, Federal University of Rio de Janeiro, Rio de Janeiro 21941-913, RJ, Brazil; robertofonseca@hucff.ufrj.br; 3 Department of Clinical Analyses and Toxicology, School of Pharmacy, Federal University of Rio de Janeiro, Rio de Janeiro 21941-599, RJ, Brazil; flaviafrattani@pharma.ufrj.br; 4 Undergraduate Program in Pharmacology, Institute of Biomedical Sciences, Federal University of Rio de Janeiro, Rio de Janeiro 21941-902, RJ, Brazil; 5 Department of BioMolecular Sciences, Division of Pharmacognosy, and Research Institute of Pharmaceutical Sciences, School of Pharmacy, University of Mississippi, Oxford, MS 38677-1848, USA

**Keywords:** anticoagulation, antithrombosis, marine glycan, sulfated fucan, sulfated galactan

## Abstract

The anticoagulant and antithrombotic properties of three structurally correlated sea urchin-derived 3-linked sulfated α-glycans and their low molecular-weight derivatives were screened comparatively through various in vitro and in vivo methods. These methods include activated partial thromboplastin time, the inhibitory activity of antithrombin over thrombin and factor Xa, venous antithrombosis, the inhibition of platelet aggregation, the activation of factor XII, and bleeding. While the 2-sulfated fucan from *Strongylocentrotus franciscanus* was observed to be poorly active in most assays, the 4-sulfated fucan from *Lytechinus variegatus*, the 2-sulfated galactan from *Echinometra lucunter* and their derivatives showed multiple effects. All marine compounds showed no capacity to activate factor XII and similar low bleeding tendencies regardless of the dose concentrations used to achieve the highest antithrombotic effect observed. The 2-sulfated galactan showed the best combination of results. Our work improves the background about the structure-function relationship of the marine sulfated glycans in anticoagulation and antithrombosis. Besides confirming the negative effect of the 2-sulfated fucose and the positive effect of the 2-sulfated galactose on anticoagulation in vitro, our results also demonstrate the importance of this set of structural requirements on antithrombosis in vivo, and further support the involvement of high-molecular weight and 4-sulfated fucose in both activities.

## 1. Introduction

Heart- and blood-related diseases, including ischaemic heart disease, deep vein thrombosis, stroke and atherosclerosis, remain one of the leading causes of death in the Western world [1]. As stated by the World Health Organization, these complications account for over one-quarter of total deaths worldwide [2]. In most of these diseases, thrombotic episodes can be managed by anticoagulant and antithrombotic medicine [3]. However, these therapies often produce undesirable and, sometimes, unavoidable side effects that range from moderate to severe [4,5,6]. Heparin, a sulfated glycan belonging to the family of the glycosaminoglycans (GAGs), is among these drugs [7]. Side effects of this complex carbohydrate include thrombocytopenia [8,9] and hemorrhagic episodes [10,11], greatly limiting its pharmacological applications. Patients undergoing heparin-based treatments must be constantly monitored throughout their entire therapy and dosages used must be controlled in real-time on the basis of observable side-effects. Hence, the development of other effective and safe anticoagulant/antithrombotic drugs remains a long-sought aim in the clinic.

Recently, novel sulfated glycans, especially those of marine origin, have been considered for their potential benefits in coagulation and thrombosis [12,13,14]. These glycans can be classified as GAGs of unique structures, such as the fucosylated chondrotin sulfates (FCSs) isolated from sea cucumbers [15,16], or as GAG mimetics, such as the sulfated fucans and sulfated galactans [12,13]. Early studies have shown that the 3-linked 2-sulfated α-galactan from the sea urchin *Echinometra lucunter*, but not the 3-linked 2-sulfated α-fucan from the sea urchin *Strongylocentrotus franciscanus*, is a thrombin (IIa) inhibitor mediated by both antithrombin (AT) or by heparin cofactor II [17]. The difference between the activities of these two polysaccharides is not very pronounced when factor Xa replaces IIa. In another work, the 2-sulfated fucose units have led to a deleterious outcome in anticoagulation while the 2,4-disulfated fucose units were observed to act as an amplifying motif for inhibition of IIa via AT [18]. A recent work describing the comparative effects of a sea urchin sulfated fucan and a sea cucumber FCS, both structurally containing 2,4-disulfated fucose units either as composing units of the backbone (former) or as lateral branching residues (latter), has reinforced the role of the 2,4-disulfated fucose motifs in coagulation and thrombosis [19]. Direct oral administration of FCS [20], or administration via gastro-resistant tablets [21], has also been recently proposed to improve the delivery, bioavailability and pharmacological function of the holothurian GAG [20,21].

**Figure 1:**
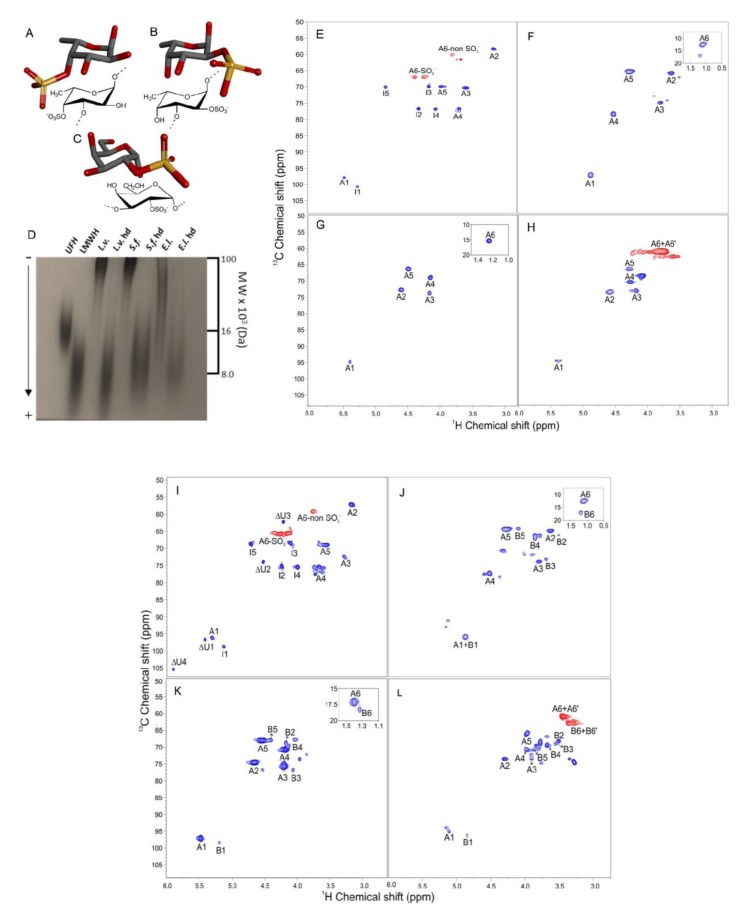
Structural representation (**A**–**C**), polyacrylamide gel electrophoresis (PAGE) (**D**) and ^1^H-^13^C dept-heteronuclear single quantum coherence (HSQC) spectra (**E**–**L**) of the three structurally correlated sea urchin sulfated glycans. Their structures are the 3-linked 4-sulfated α-fucan from *Lytechinus variegatus* (**A**), the 3-linked 2-sulfated α-fucan from *Strongylocentrotus franciscanus* (**B**), and the 3-linked 2-sulfated α-galactan from *Echinometra lucunter* (**C**). The molecular weight (MW) ranges of the native sulfated glycans, of their low-MW derivatives and of the standards unfractionated heparin (UFH) (MW~16 kDa) and low molecular-weight heparin (LMWH) (MW ~ 8 kDa) are comparatively analyzed through PAGE (**D**). The ^1^H-^13^C dept-HSQC spectra were recorded for UFH (**E**), *L.v* (**F**), *S.f.* (**G**), *E.l.* (**H**), LMWH (**I**), *L.v.* hd (**J**), *S.f.* hd (**K**), and *E.l.* hd (**L**). While cross-peaks from CH and CH_3_ (in-phase) are shown in blue, those from CH_2_ (anti-phase) are shown in red. In panels (**E**) and (**I**), peaks labeled with (**A**) and (**I**) stand for glucosamine and iduronate, respectively. In panel (**F**–**H**) and(**J**–**L**), (**A**) stands for the sulfated units and (**B**) stands for the desulfated units.

Despite a growing number of data and publications on the potential anticoagulant and antithrombotic effects of marine sulfated glycans, studies concerning the potential antithrombotic actions of sea urchin-derived sulfated glycans examined through in vivo methods have not been extensively carried out. Here, we examine the anticoagulant and antithrombotic properties of three sea urchin-derived sulfated α-glycans via a complementary in vitro and in vivo experimental series that includes (a) activated partial thromboplastin time (aPTT), (b) the inhibitory activity of AT over the blood factor thrombin (IIa), (c) in vivo venous antithrombosis, (d) the inhibition of platelet aggregation, (e) the activation of factor XII, and (f) bleeding tendency. The sea urchin sulfated glycans are the 3-linked 4-sulfated α-fucan isolated from *Lytechinus variegatus* [22] (*L.v.*) (Figure 1A), the 3-linked 2-sulfated α-fucan from *S. franciscanus* [23] (*S.f.*) (Figure 1B), and the 3-linked 2-sulfated α-galactan from *E. lucunter* [24] (*E.l.*) (Figure 1C). These three sea urchin sulfated glycans are structurally correlated. Among pairs of species, all structural features are the same, except one. For example, *L.v.* is 4-sulfated and *S.f.* is 2-sulfated. While *S.f.* is composed of fucose, *E.l.* is composed of galactose. The combined use of these three molecules enables an advanced investigation of structure-activity relationships (SAR), especially in light of the demonstrated impact of sulfation positions (C4 versus C2) and monosaccharide compositions (fucose versus galactose) on anticoagulant and antithrombotic effects. To enlarge this current SAR study, we have also examined the impact of molecular weights (MWs) of these glycans by testing their low MW derivatives produced by mild acid hydrolysis. The gold standard therapeutics, unfractionated heparin (UFH) and low molecular-weight heparin (LMWH) were employed as controls in all assays performed. Our work improves the current background about the SAR of the marine sulfated glycans in anticoagulation and antithrombosis. Besides confirming the negative effect of the 2-sulfated fucose and the positive effect of the 2-sulfated galactose on anticoagulation in vitro, the results here obtained also show the importance of this set of structural characteristics for in vivo antithrombosis, and further demonstrate the involvement of the high-MW and the 4-sulfated fucose in both medicinal activities.

## 2. Results and Discussion

### 2.1. Compounds and Structural Integrity

Figure 1E–L displays fully assigned nuclear magnetic resonance (NMR) ^1^H-^13^C distortionless enhancement by polarization transfer (dept)-heteronuclear single quantum coherence (HSQC) spectra of all eight compounds studied in this work. These compounds are the following: the native 3-linked 4-sulfated α-fucan from *L. variegatus* (*L.v.*), its low MW derivative (*L.v.* hd), the native 3-linked 2-sulfated α-fucan from *S. franciscanus* (*S.f.*), its low MW derivative (*S.f.* hd), the native 3-linked 2-sulfated α-galactan from *E. lucunter* (*E.l.*), its low MW derivative (*E.l.* hd), and the heparin controls UFH and LMWH. The sea urchin low-MW derivatives were prepared by mild acid hydrolysis (Figure S1) as reported previously [25,26]. While *L.v.* hd, was obtained with 3 h of hydrolysis with 0.04 M HCl, *S.f.* hd was generated with 4 h of hydrolysis with 0.4 M HCl and *E.l.* hd, with 8 h of hydrolysis using 1.0 M HCl, all at same temperature of 60 °C (Figure S1) [25,26].

The MWs of these derivatives are ranged closely to the oligosaccharide distribution of the LMWH (Figure 1D). Besides the current analysis by polyacrylamide gel electrophoresis (PAGE) of the derivatives, as illustrated in Figure 1D, more detailed analyses obtained by an ultra-fast liquid chromatography (UFLC) system were also obtained and presented in our previous works (see references [25,26] for the UFLC data). The combination of PAGE and UFLC analyses increases our confidence to safely state that the MW distribution of the sea urchin sulfated glycan-derived oligosaccharides produced by mild acid hydrolysis ranges close to that from the LMWH sample, which is from ~3 to ~8 kDa. The MWs of the native polymers vary considerably and are highly polydisperse, as seen in the PAGE analysis in Figure 1D, but always above ~15 kDa and with the major components above ~100 kDa.

Since the structures of all glycans studied in this work are known, the ^1^H-^13^C dept-HSQC spectra displayed at Figure 1E–L were recorded not for elucidation purposes, but primarily for checking the structural integrity and assessing some possible chemical variations such as polymeric homogeneity and sulfation degrees, besides evaluating the levels of purity in the analyzed samples to be further used in the in vitro anticoagulant and in vivo antithrombotic assays. As expected, the values of ^1^H and ^13^C chemical shifts are all in accordance with the previous publications (Table S1). The desulfation rates of the low-MW derivatives were calculated based on the relative intensities of the anomeric ^1^H-^13^C cross-peak in the sulfated units (labeled as A1 at the downfield region of the spectra) and in the desulfated unit (labeled as B1 on the upfield region of the spectra; see the ^1^H-^13^C dept-HSQC spectra of the depolymerized products, Figure 1J for *L.v.* hd, Figure 1K for *S.f.* hd, and Figure 1L for *E.l.* hd). The sulfation/desulfation ratios of *L.v.* hd, *S.f.* hd and *E.l.* hd were, respectively, 80/20, 90/10, and 60/40.

From the analysis of the ^1^H-^13^C dept-HSQC spectra, the structural integrity of all eight sulfated glycans was ensured, and the following structural compositions were securely confirmed for each compound: UFH and LMWH are mainly composed of the trisulfated disaccharide unit [→4)-α-iduronate(2SO_3_^−^)-(1→4)-α-glucosamine(NSO_3_^−^6SO_3_^−^)-(1→]_n_ (Figure 1E). In addition to this major composition, LMWH additionally contains 4,5-unsaturated uronic acid (ΔU) in the terminal nonreducing-end residues, as seen with the ^1^H-^13^C cross-peaks assigned with Δ such as Δ1 with δ_H/C_ at 5.5/96.8 ppm and Δ4 δ_H/C_ at 6.0/105.3 ppm (Figure 1I). *L.v.* is composed of [→3)-α-fucose(4SO_3_^−^)-(1→]_n_ (Figure 1A). *S.f.* is composed of [→3)-α-fucose(2SO_3_^−^)-(1→]_n_ (Figure 1B). *E.l.* is composed of [→3)-α-galactose(2SO_3_^−^)-(1→]_n_ (Figure 1C). *L.v.* hd is composed of 80% [→3)-α-fucose(4SO_3_^−^)-(1→]_n_ and 20% [→3)-α-fucose-(1→]_n_ (Figure 1J). *S.f.* hd is composed of 90% [→3)-α-fucose(2SO_3_^−^)-(1→]_n_ and 10% [→3)-α-fucose-(1→]_n_ (Figure 1K). *E.l.* hd is composed of 60% [→3)-α-galactose(2SO_3_^−^)-(1→]_n_ and 40% [→3)-α-galactose-(1→]_n_ (Figure 1L). Based on more detailed NMR analyses, principally those from nuclear Overhauser effect (NOESY) spectra in our previous works [25,26], we have also identified that the sulfated and desulfated units of *L.v.* hd, *S.f.* hd and *E.L.* hd happen randomly throughout the backbone of the medium-sized oligosaccharides and do not form clearly defined domains in their chains. This confirmation comes from the lack of clear NOE cross-peaks between the sulfated (assigned as A) and the desulfated (assigned as B) units [25,26].

### 2.2. Anticoagulation

**Figure 2:**
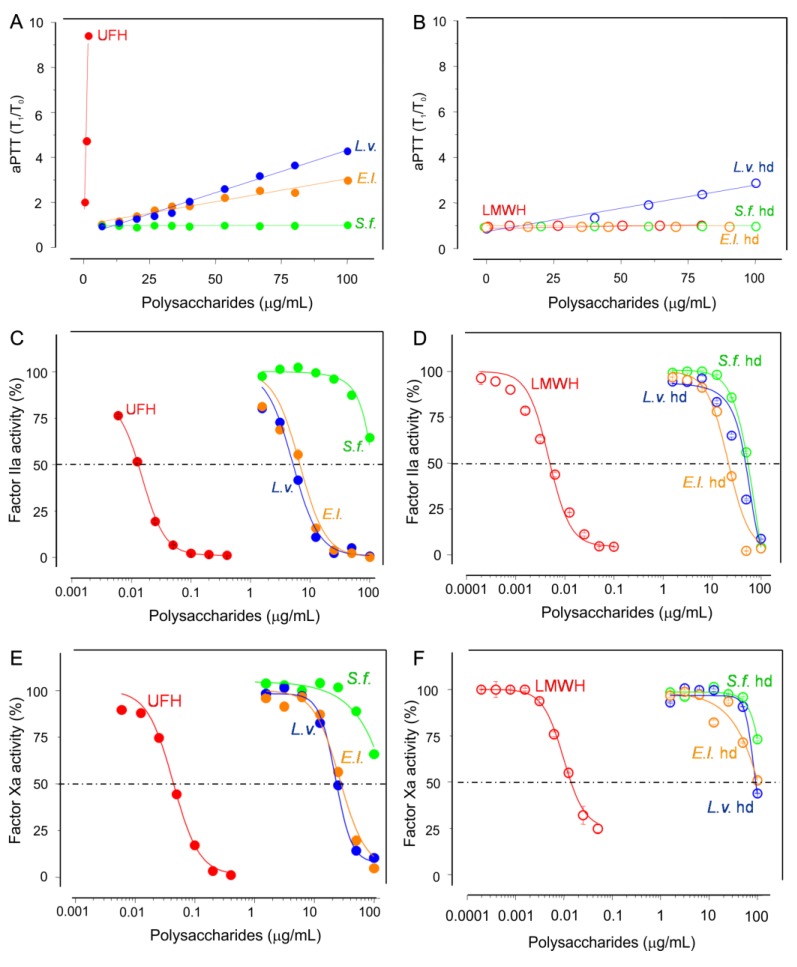
Anticoagulant properties of the native sea urchin sulfated glycans (**A**,**C**,**E**) and their low-MW derivatives (**B**,**D**,**F**) screened by aPTT (**A**,**B**) and by AT-mediated IIa (**C**,**D**) and Xa (**E**,**F**) inhibition.

The anticoagulant effects of the native sea urchin sulfated glycans (Figure 2A,C,D) and their low-MW derivatives (Figure 2B,D,F) were examined by two principal approaches. The first approach is the aPTT, a less specific assay generally used to screen the ability of molecules in their capacities to retard the coagulation time (Figure 2A,B). In the aPTT assay, the potential anticoagulant activities of compounds are usually tested in regular plasma in which all blood coagulant (co)factors are normally present altogether. The second approach involves purified coagulation factors (panels C–F in Figure 2). In this approach, the potential anticoagulant effects of the sulfated glycans were examined in light of their capacities to catalyze the inhibitory function of certain serpins, such as AT, over their common targets such as the blood protease factors IIa (Figure 2C,D) and Xa (Figure 2E,F).

In the aPTT assay, *L.v.*, *E.l.* and *L.v.* hd were the most active compounds among the six tested marine sulfated sugars (Figure 2A,B). Nonetheless, they were not as potent as the control UFH. As seen, while the activity of UFH reached easily the T_1_/T_0_ value of 10 with the dose of 10 μg/mL (red curve in Figure 2A), the most active sea urchin sulfated glycan, *L.v.*, only reached the T_1_/T_0_ value of 4 with a dose ten times more concentrated (blue curve in Figure 2A). Interestingly, the low-MW derivative of this sulfated fucan, *L.v.* hd, still retained some of the anticoagulant effect, albeit with slightly lower potency than the native compound. Compared to *L.v.*, *L.v.* hd hardly reached the T_1_/T_0_ value of 3 with the dose of 100 μg/mL (blue curve in Figure 2B).

Regarding the AT-mediated anti-IIa and anti-Xa activities, *S.f.* was observed to be poorly active whereas *L.v.* and *E.l.* were more effective, showing similar potencies of inhibition in both proteases (Figure 2C,E and Table 1). In terms of UI·mg^−1^, both *L.v.* and *E.l.* showed nearly equal effects in the narrow range of 0.4–0.5 and 0.3 for their respective anti-IIa and anti-Xa properties (Table 1). This set of data clearly indicates equal potencies for both *L.v.* and *E.l.* samples in both systems. Upon reduction of the MW, the loss of the anti-IIa activity of *L.v.* and *E.l.* were, respectively, around 6- and 4-fold lower (Figure 2C versus Figure 2D), and, respectively, around 5- and 3-fold lower for the anti-Xa activities, as interpreted in terms of UI·mg^−1^ values (Figure 2E versus Figure 2F). *L.v.* hd, *E.l.* hd, *S.f.* and its low MW derivative, *S.f.* hd, all presented no marked capacity for to enhance AT inhibitory function over IIa and Xa. From this set of results, two findings were re-observed for the in vitro anticoagulant activity of the sea urchin-derived sulfated glycans: (a) 2-*O*-sulfation on fucose units of 3-linked α-glycans has a negative impact, as indicated previously [17,18,27], and (b) a high-MW of the native compound is essential to the final outcome, as suggested previously [28]. One new finding was also introduced from our in vitro study, and that concerns the positive effect of the 4-*O*-sulfated fucose units on the sea urchin-derived 3-linked sulfated α-glycans.

### 2.3. Antithrombosis

**Figure 3:**
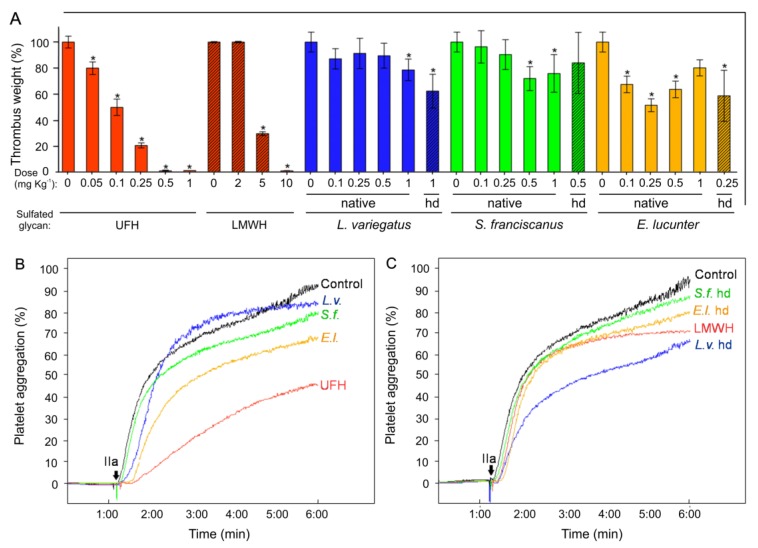
Antithrombotic properties of the native sea urchin sulfated glycans (**A**,**B**) and their low-MW derivatives (**C**), in venous model of thrombosis, (**A**) and platelet aggregation (**B**,**C**).

The potential antithrombotic properties of the three sea urchin sulfated α-glycans and their low-MW derivatives were examined by the in vivo venous thrombosis assay using thromboplastin as the thrombogenic stimulus (Figure 3A). The native compounds were assayed in a similar dose window as the UFH. The most active dose of the native sulfated glycans was selected to be used further for their low-MW derivatives, analyzed comparatively in the same dose window of LMWH. Using this in vivo model and experimental rationale, we have observed that all six sea urchin-derived compounds have presented activity in a dose–response fashion, despite their lower efficacy as compared to the heparin standards (Figure 3A). Although UFH and LMWH can prevent 100% of thrombus formation with the doses of 0.5 and 10 mg·kg^−1^, respectively; *L.v.*, *S.f.* and *E.l.* presented the capacity to inhibit 20%, 25% and 50% of the thrombus weight at the doses of 1.0, 0.5 and 0.25 mg·kg^−1^, respectively.

Interestingly, the low-MW derivatives of all three sea urchin sulfated glycans were also very active in the venous antithrombotic model (hatched bars in Figure 3A). *L.v.* hd, *S.f.* hd and *E.l.* hd inhibited respectively 37.6%, 16% and 41.2% of the thrombus weight with the most active dose achieved by their native counterparts. Although the percentage of thrombus inhibition of *L.v.* hd seemed apparently higher than its native molecule tested at the same dosage, the difference is not statistically significant. Among all six sea urchin-derived sulfated glycans screened in the in vivo antithrombosis experiment, *E.l.* was the most efficacious and potent (yellow bars in Figure 3A).

Intriguing observations could be raised from the in vivo antithrombotic experiment. Firstly, it is clear to see the stronger effects of *L.v.* and *L.v.* hd (both at 1 mg·kg^−1^), *S.f.* (0.5 and 1 mg·kg^−1^), *S.f.* hd (0.5 mg·kg^−1^) and *E.l.* and *E.l.* hd (at all tested doses) as compared to the LMWH standard at the dose of 2 mg·kg^−1^. Secondly, the *E.l.* has a dual antithrombotic behavior. At the doses of 0.1 and 0.25 mg·kg^−1^, this sulfated galactan exhibits higher effects. And as the doses increase, its antithrombotic property is reduced. This dual effect has been observed previously for the sulfated galactan from *Botryocladia occidentalis* [28,29] and highlighted recently in a review article dedicated to this type of phenomenon [30]. Probably, at higher doses than 0.25 mg·kg^−1^, the sulfated glycan *E.l.* is making complexes with the coagulation factors (proteases and inhibitors) with a different stoichiometry as compared to the lower doses. Future analysis must be carried out in order to understand this process in more details. Thirdly, it is interesting to see that the *E.l.* hd presents nearly equal activity to the native molecule tested at the same dose.

### 2.4. Platelet Aggregation

The six compounds were further examined as inhibitors of the in vitro platelet aggregation (Figure 3B,C) as compared to the two heparins. Three final concentrations (10, 50 and 150 μg·mL^−1^) were evaluated. These doses were chosen based on the range of activity observed for the AT-dependent anti-IIa and anti-Xa activities (panels C–D in Figure 2). Similar curves were observed in all three concentrations. The curves obtained with the dose of 150 μg·mL^−1^ were then selected for illustration in Figure 3B,C. IIa was used as an agonist in the assay and the differential activity of the eight samples were monitored over 5 min. The most active compounds were the native *E.l.* (yellow curve in Figure 3B) and *L.v.* hd (blue curve in Figure 3C). Both compounds inhibited approximately 30% of the platelet aggregation at the end of the experimental period as compared to the curves seen for the UFH and LMWH controls, whose inhibitory activities were, in turn, approximately 55% and 30%, respectively (Figure 3B,C). The effect of *E.l.* in venous thrombosis (yellow bars in Figure 3A), and of *E.l.* and *L.v.* hd in platelet aggregation inhibition (yellow and blue curves in Figure 3B and Figure 3C, respectively) coincide somewhat with the results seen in the anticoagulant assays (Figure 2), supporting the notion of multiple and different effects in the anticoagulant and antithrombotic systems.

### 2.5. Potential Side Effects

**Figure 4:**
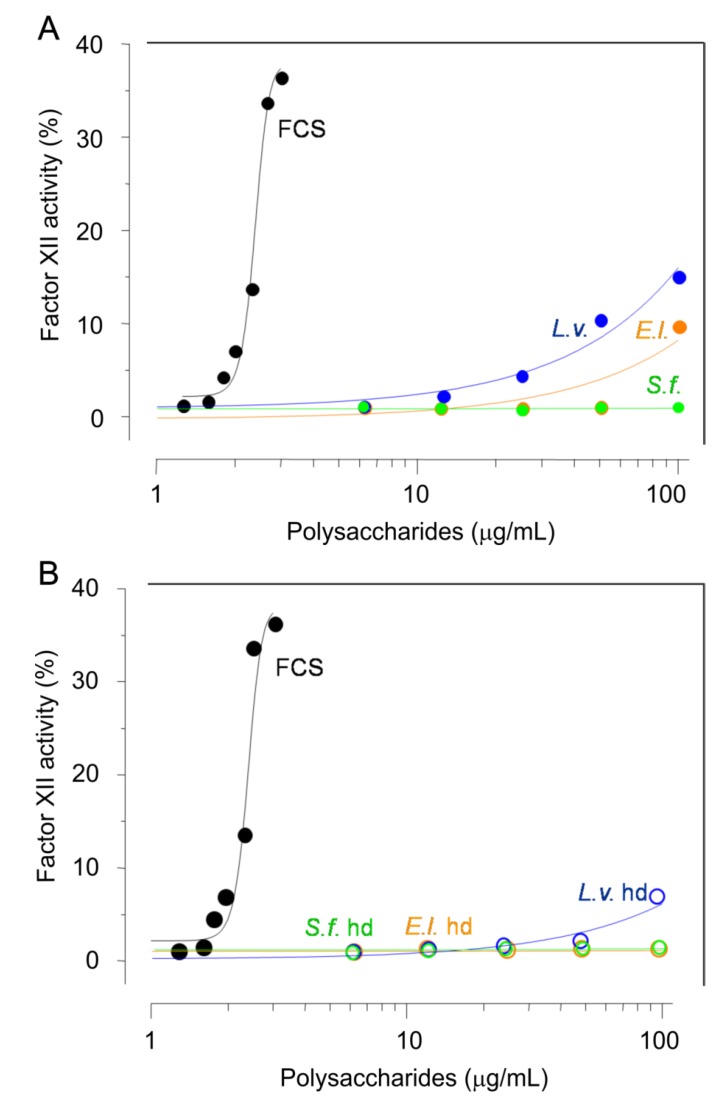

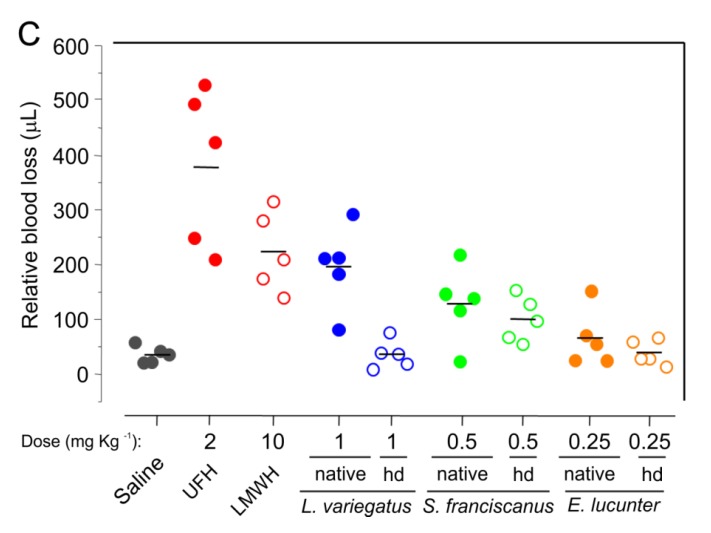
Effects of the native sea urchin sulfated glycans (**A**, and solid circles in panel **C**) and low-MW derivatives (**B**, and open circles in panel **C**) in assays of factor XII activation (**A**,**B**) and bleeding tendency (**C**).

Two potential undesirable effects of sulfated glycans in antithrombosis are (i) the activation of factor XII because of the presence of negatively charged surfaces which could, in turn, lead to generation of bradykinin and subsequent hypotension, and (ii) the bleeding risk because of the enhanced or uncontrolled anticoagulant/antithrombotic effects in the body. Therefore, we aimed to examine these two potential effects on both the native sea urchin sulfated glycans and their low-MW derivatives through in vitro and in vivo methods. The curves in Figure 4A,B depict the induction degrees of factor XII activation, respectively for the native and for the low-MW derivatives. Curves were compared to the degrees of activity of the FCS isolated from the sea cucumber *Ludwigothurea grisea.* This FCS was employed here as positive control because of its ability to activate factor XII [31]. From panels shown in Figure 4, it is clear to note that all six sea urchin-derived sulfated carbohydrates are devoid of such effect.

To evaluate the hemorrhagic risk, a bleeding time model was employed (Figure 4C). In this experiment, the most active doses of the sulfated glycans seen on the in vivo venous thrombosis experiment (Figure 3A) were tested. It is clear to see that the marine sulfated glycans showed lower bleeding effects (Figure 4C) as compared to the heparins, regardless of the different doses analyzed. The standards UFH and LMWH were observed very hemorrhagic (Figure 4C) at the tested doses. Among all six marine carbohydrates, *L.v.* presented the highest blood loss (closed blue circles in Figure 4C). From the set of results obtained in Figure 4, we can conclude that the sea urchin-derived sulfated glycans and their low-MW derivatives have no capacity to induce factor XII activation and low hemorrhagic propensity at their most active antithrombotic doses observed in this study.

## 3. Materials and Methods

### 3.1. Purification of the Sea Urchin Sulfated Glycans

The purification of the *L.v.*, *S.f.* and *E.l.* was performed as described previously [17,22].

### 3.2. Production of the Low-MW Derivatives

The low-MW derivatives with a close MW range of LMWH from the three structurally correlated sea urchin sulfated glycans were produced by acid hydrolysis, as recently described [25,26].

### 3.3. PAGE

The native and low-MW derivatives of the sea urchin sulfated glycans (~10 µg of each material) were loaded to a 6.0% 1-mm thick polyacrylamide gel slab in 0.06 M Tris-HCl (pH 8.6) and run for ~60 min at 100 V. After the electrophoresis, the samples were stained with 0.1% toluidine blue in 1% acetic acid, and washed for about 2 h in 1% acetic acid. The MWs were estimated by comparison with the standard compounds used as molecular markers, as previously described [32]. The standards used were chondroitin 4-sulfate from bovine trachea (~40 kDa), UFH (~16 kDa) and LMWH (~8.0 kDa). Sample concentrations were the same used for all sulfated glycans.

### 3.4. NMR ^1^H-^13^C Dept-HSQC

All NMR ^1^H-^13^C dept-HSQC spectra were recorded on a Bruker DRX 600 MHz instrument. About 3 mg of each sample were dissolved in 0.5 mL of 99.9% deuterium oxide. All spectra were recorded at 298 K with HOD suppression by pre-saturation. ^1^H-^13^C dept-HSQC spectra were run with 1024 × 256 points and globally optimized alternating phase rectangular pulses for decoupling. Chemical shifts are relative to external trimethylsilylpropionic acid at 0 ppm for ^1^H and to methanol for ^13^C.

### 3.5. Activated Partial Thromboplastin Time (aPTT)

The aPTT assay was performed according to the manufacturer’s specifications (Biolab-Merieux AS, Rio de Janeiro, Brazil). The results are expressed as ratios of clotting time in the presence (T_1_) or absence (T_0_) of sulfated glycan.

### 3.6. Inhibition of Thrombin or Factor Xa by Antithrombin in the Presence of Sulfated Glycans

Incubations were performed in 96-well plates. The final concentrations of the reactants were 10 nM antithrombin, 2 nM thrombin or factor Xa, and 0–100 μg/mL sulfated glycans in 40 μL of TS/PEG buffer (0.02 M Tris/HCl, 0.15 M NaCl, and 1.0 mg/mL polyethylene glycol 8000, pH 7.4). Thrombin or factor Xa was added lastly to initiate the reaction. After incubation for 60 s, at 37 °C, 25 μL of chromogenic substrate (0.4 mM) S-2238 for thrombin or S-2222 for factor Xa (Chromogenix AB, Mondal, Sweden) was added, and the absorbance at 405 nm recorded for 300 s (Plate reader Thermo-max, America Devices, Sunnyvale, CA, USA). The rate of change of the absorbance was proportional to the amount of thrombin or factor Xa activity remaining. Thrombin activity was defined as 100% in control samples lacking sulfated glycans. The heparin used as positive control was the 6th International Standard (200 IU/mg) from the National Institute for Biological Standards and Control (Potters Bar, UK).

### 3.7. Venous Thrombosis

Antithrombotic activity was investigated in rats with rabbit brain thromboplastin as the thrombogenic stimulus [33]. The experiments were performed following the recommendations of animal care defined by the Institutional Committee (Institute of Medical Biochemistry, Federal University of Rio de Janeiro, Brazil). We followed the institutional guidelines for animal care and experimentation. Rats (both sexes, five animals per dose) were anesthetized with intramuscular injection of 100 mg/kg body weight of ketamine (Cristália, São Paulo, Brazil) and 16 mg/kg body weight of xylazine (Bayer AS, São Paulo, Brazil). The inferior vena cava was isolated and different doses of GAGs were infused and allowed to circulate for 5 min. Different doses of sulfated glycans were infused into the right carotid artery and allowed to circulate for 5 min. The inferior vena cava was isolated and brain thromboplastin (5 mg/kg body weight) from Biolab-Merieux AS (Rio de Janeiro, Brazil) was slowly injected intravenously. After 1 min, 1.0 cm of isolated vena cava was clamped off using distal and proximal sutures. After a 20 min stasis, the thrombus formed inside the occluded segment was carefully removed, washed with phosphate-buffered saline, dried for 1 h at 60 °C, and weighed. Mean thrombus weight was obtained by the average weight from each group and then expressed as the percentage of the thrombus weight in the absence of sulfated glycan administration.

### 3.8. Platelet Aggregation

Peripheral venous blood was drawn from human volunteers aged 20–25 years who were non-smokers and reported having not taken any drug during the previous 10 days. (Certificate of Presentation for Ethical Appreciation CAAE 60160716.3.0000.5257). Blood was collected into tubes containing 3.8% sodium citrate and centrifuged at 200× *g* for 10 min, at room temperature, to obtain platelet-rich plasma (PRP). An aliquot of PRP was further centrifuged at 1200× *g* for 10 min to obtain platelet-poor plasma (PPP). Platelets were then washed twice with calcium-free Tyrode’s buffer, pH 6.5, containing 0.1% glucose, 0.2% gelatin, 0.14 M NaCl, 0.3 M NaHCO_3_, 0.4 mM NaH_2_PO_4_, 0.4 mM MgCl_2_, 2.7 mM KCl, and 0.2 mM EGTA. Platelet aggregation in PRP was measured by a turbidimetric method (final platelet count was 300,000/μL) on a Chronolog Aggregometer (Havertown, PA, USA). Baseline (0%) and 100% aggregation were established by measuring the light transmission through PRP and PPP, respectively. Assays were performed at 37 °C using a Chronolog Aggregometer (Havertown, PA, USA). Sulfated glycans were incubated for 1 min in 350 μL of washed platelet suspensions containing 4.4 μL NaCl (1 M) at 3 different final concentrations (10, 50 and 150 μg/mL). Aggregation was then triggered by addition of 5 μL IIa for 10 nM final concentration. This concentration of IIa was the lowest to induce complete platelet aggregation with saline as control. The levels of aggregation for the different samples were recorded during 5 min, right after the addition of the agonist. Controls without sulfated glycans (just saline) were tested at the same time. The samples containing the sulfated glycans were therefore assayed in triplicate because of the 3 different concentrations. The resultant curves were very similar for all 3 concentrations.

### 3.9. Bleeding

Wistar rats (both sexes) were anesthetized with a combination of xylazine and ketamine. A cannula was inserted into the right carotid artery for administration of different doses of sulfated polysaccharides. After the sample had circulated for 5 min, the tail was cut 3 mm from the tip and carefully immersed in 40 mL distilled water, at room temperature. Blood loss after 60 min was determined by measuring the haemoglobin dissolved in the water using bya spectophotometric method, as described previously [34]. The volume of blood was deduced from a standard curve based on A 540 nm. Modified from Fonseca et al. [35].

### 3.10. Activation of Factor XII

Factor XII activation assays were carried out in 96-well plates. Normal human plasma was diluted with three volumes of TS buffer and samples (40 μL) were incubated with different concentrations of sulfated glycans (30 μL). After incubation for 60 s at 37 °C, 30 μL of 0.3 mM chromogenic substrate S-2302 (Chromogenix AB) was added, and the absorbance at λ = 405 nm was recorded for 300 s (Plate reader Thermo-max, America Devices). Full activation of factor XII was achieved in 300 s [29,31] No activation occurred in control experiments in the absence of sulfated glycans. The positive control used was FCS from *L. grisea* which exhibits significant activity on activation of factor XII, as reported previously [31].

## 4. Conclusions

An ideal anticoagulant/antithrombotic agent should exert multiple combined properties, including catalytic activity on the inhibitory process of serpins (mainly AT) over blood factors (especially IIa and Xa), inhibitory action on platelet aggregation, the absence of factor XII activation and a low bleeding risk. Heparin is the most frequently used drug in thromboembolic diseases; however, it presents many downsides including high hemorrhagic risk. Other sulfated glycans have been recently investigated regarding their actions in the systems of coagulation and thrombosis. Here, we have performed various in vitro anticoagulant and in vivo antithrombotic assays on three structurally correlated sea urchin-derived 3-linked sulfated α-glycans and their low-MW derivatives. The 2-sulfated fucan from *S. franciscanus* was observed to be poorly active in most assays. The conformational preference for interaction with AT was proposed by Becker et al. in a previous work which aimed to understand the absence of the anticoagulant activity of this 2-sulfated fucan [36]. The 4-sulfated fucan from *L. variegatus*, the 2-sulfated galactan from *E. lucunter* and the low-MW derivative from the *L. variegatus* presented multiple effects. All marine sulfated glycans (both native and derivatives) showed low bleeding tendencies and no capacity to induce the activation of factor XII. In all, the 2-sulfated galactan demonstrated the best conjunction of properties, particularly considering its inhibitory effects in the in vitro coagulation assays (yellow curves in panels A, C and D of Figure 2), in the in vivo thrombosis assay (yellow bars in Figure 3A), and in the in vitro assay of platelet aggregation (yellow curve of Figure 3B). A high-MW, as naturally found in the native molecules, is required for achieving the best result.

Overall, our study has clearly demonstrated that the structurally defined and correlated sea urchin sulfated glycans can serve as useful molecular tools to unveil both the underlying mechanisms of action and the structural requirements of the sulfated glycans in anticoagulation and antithrombosis. Considering the observed SAR, our data have not only confirmed the negative impact of the 2-sulfated fucose units and the positive action of the 2-sulfated galactose unit during the in vitro anticoagulant activity, as previously reported [17,18,27], but also introduced this same set of requirements to the in vivo antithrombotic activity. Three-linked sulfated α-fucans containing 2-sulfated fucose units from other sea urchin species such as *Strongylocentrotus pallidus* and *Arbacia lixula* were also tested previously, and their low anticoagulant activities were associated to the content of this unit, confirming therefore the negative impact of the 2-sulfated fucose [18]. This work further demonstrates the importance of the high-MW native molecules and 4-sulfated fucose on in vitro and in vivo actions. Together, the results here described reinforce the notion that there are indeed structural requirements on the marine sulfated glycans for their controlling functions over coagulation factors. These include the positions of sulfate groups, sugar compositions, and chain lengths. These features contribute more specifically to the clinical outcome than the overall net charge of the polysaccharide. However, it is worth mentioning that these chemical features and their consequent medical effects may be altered by metabolic processing in the gastrointestinal tract during oral administration. Hence, further investigations are required in order to confirm whether the MWs and sulfation patterns of these marine molecules will be maintained, keeping also their clinical activities if orally absorbed.

## Figures and Tables

**Table 1:** Potency values (UI/mg) from the in vitro anticoagulant (AT-mediated anti-IIa and anti-Xa) assays of the sea urchin sulfated glycans and their low-MW derivatives.

Glycan	AT/IIa	AT/Xa
UFH	210	204
LMWH	35	100
*L.v.*	0.44	0.29
*L.v.* hd	0.07	0.06
*S.f.*	0.02	0.05
*S.f.* hd	0.05	0.04
*E.l.*	0.56	0.27
*E.l.* hd	0.12	0.08

## Supplementary Materials

The supplemental material is available at http://www.mdpi.com/1660-3397/16/9/304/s1.

## References

[1] Raskob G.E., Angchaisuksiri P., Blanco A.N., Buller H., Gallus A., Hunt B.J., Hylek E.M., Kakkar A., Konstantinides S.V., McCumber M. (2014). Thrombosis: A major contributor to global disease burden. Arterioscler. Thromb. Vasc. Biol..

[2] The Top 10 Causes of Death. http://www.who.int/mediacentre/factsheets/fs310/en/.

[3] Spyropoulos A.C. (2008). Brave new world: The current and future use of novel anticoagulants. Thromb. Res..

[4] Mannucci P.M., Franchini M. (2011). Old and new anticoagulant drugs: A minireview. Ann. Med..

[5] Fanikos J., Cina J.L., Baroletti S., Fiumara K., Matta L., Goldhaber S.Z. (2007). Adverse drug events in hospitalized cardiac patients. Am. J. Cardiol..

[6] Moore T.J., Cohen M.R., Furberg C.D. (2007). Serious adverse drug events reported to the Food and Drug Administration, 1998–2005. Arch. Intern. Med..

[7] Mulloy B., Hogwood J., Gray E., Lever R., Page C.P. (2016). Pharmacology of heparin and related drugs. Pharmacol. Rev..

[8] Ahmed I., Majeed A., Powell R. (2007). Heparin induced thrombocytopenia: Diagnosis and management update. Postgrad. Med. J..

[9] Baroletti S.A., Goldhaber S.Z. (2006). Heparin-induced thrombocytopenia. Circulation.

[10] Ramirez-Lassepas M., Quinones M.R. (1984). Heparin therapy for stroke: Hemorrhagic complications and risk factors for intracerebral hemorrhage. Neurology.

[11] Clark W.M., Madden K.P., Lyden P.D., Zivin J.A. (1991). Cerebral hemorrhagic risk of aspirin or heparin therapy with thrombolytic treatment in rabbits. Stroke.

[12] Pomin V.H. (2012). Fucanomics and galactanomics: Current status in drug discovery, mechanisms of action and role of the well-defined structures. Biochim. Biophys. Acta.

[13] Pomin V.H. (2015). Marine non-glycosaminoglycan sulfated glycans as potential pharmaceuticals. Pharmaceuticals.

[14] Pomin V.H. (2015). A dilemma in the glycosaminoglycan-based therapy: Synthetic or naturally unique molecules?. Med. Res. Rev..

[15] Pomin V.H. (2014). Holothurian fucosylated chondroitin sulfate. Mar. Drugs.

[16] Pomin V.H. (2015). Medical gains of chondroitin sulfate upon fucosylation. Curr. Med. Chem..

[17] Pereira M.S., Vilela-Silva A.C.E.S., Valente A.P., Mourão P.A.S. (2002). A 2-sulfated, 3-linked α-L-galactan is an anticoagulant polysaccharide. Carbohydr. Res..

[18] Pereira M.S., Melo F.R., Mourão P.A.S. (2002). Is there a correlation between structure and anticoagulant action of sulfated galactans and sulfated fucans?. Glycobiology.

[19] Fonseca R.J.C., Santos G.R.C., Mourão P.A.S. (2009). Effects of polysaccharides enriched in 2,4-disulfated fucose units on coagulation, thrombosis and bleeding. Practical and conceptual implications. Thromb. Haemost..

[20] Fonseca R.J.C., Mourão P.A.S. (2006). Fucosylated chondroitin sulfate as a new oral antithrombotic agent. Thromb. Haemost..

[21] Fonseca R.J.C., Sucupira I.D., Oliveira S.N.M.C.G., Santos G.R.C., Mourão P.A.S. (2017). Improved anticoagulant effect of fucosylated chondroitin sulfate orally administered as gastro-resistant tablets. Thromb. Haemost..

[22] Cinelli L.P., Castro M.O., Santos L.L., Garcia C.R., Vilela-Silva A.C.E.S., Mourão P.A.S. (2007). Expression of two different sulfated fucans by females of *Lytechinus variegatus* may regulate the seasonal variation in the fertilization of the sea urchin. Glycobiology.

[23] Vilela-Silva A.C.E.S., Alves A.P., Valente A.P., Vacquier V.D., Mourão P.A.S. (1999). Structure of the sulfated alpha-L-fucan from the egg jelly coat of the sea urchin *Strongylocentrotus franciscanus*: Patterns of preferential 2-*O*- and 4-*O*-sulfation determine sperm cell recognition. Glycobiology.

[24] Alves A.P., Mulloy B., Diniz J.A., Mourão P.A.S. (1997). Sulfated polysaccharides from the egg jelly layer are species-specific inducers of acrosomal reaction in sperms of sea urchins. J. Biol. Chem..

[25] Queiroz I.N.L., Wang X., Glushka J.N., Santos G.R.C., Valente A.P., Prestegard J.H., Woods R.J., Mourão P.A.S., Pomin V.H. (2015). Impact of sulfation pattern on the conformation and dynamics of sulfated fucan oligosaccharides as revealed by NMR and MD. Glycobiology.

[26] Queiroz I.N.L., Vilela-Silva A.C.E.S., Pomin V.H. (2016). Oligosaccharides from the 3-linked 2-sulfated alpha-L-fucan and alpha-l-galactan show similar conformations but different dynamics. Glycobiology.

[27] Pomin V.H. (2014). Anticoagulant motifs of marine sulfated glycans. Glycoconj. J..

[28] Quinderé A.L., Santos G.R., Oliveira S.N., Glauser B.F., Fontes B.P., Queiroz I.N., Benevides N.M., Pomin V.H., Mourão P.A. (2014). Is the antithrombotic effect of sulfated galactans independent of serpin?. J. Thromb. Haemost..

[29] Melo F.R., Mourão P.A. (2008). An algal sulfated galactan has an unusual dual effect on venous thrombosis due to activation of factor XII and inhibition of the coagulation proteases. Thromb. Haemost..

[30] Pomin V.H. (2016). Dual and antagonic therapeutic effects of sulfated glycans. Bioorg. Med. Chem..

[31] Fonseca R.J.C., Oliveira S.N.M.C.G., Pomin V.H., Mecawi A.S., Araujo I.G., Mourão P.A.S. (2010). Effects of oversulfated and fucosylated chondroitin sulfates on coagulation. Challenges for the study of anticoagulant polysaccharides. Thromb. Haemost..

[32] Pomin V.H., Pereira M.S., Valente A.P., Tollefsen D.M., Pavão M.S., Mourão P.A.S. (2005). Selective cleavage and anticoagulant activity of a sulfated fucan: Stereospecific removal of a 2-sulfate ester from the polysaccharide by mild acid hydrolysis, preparation of oligosaccharides, and heparin cofactor II-dependent anticoagulant activity. Glycobiology.

[33] Wessler S., Reimer S.M., Sheps M.C. (1959). Biologic assay of a thrombosis-inducing activity in human serum. J. Appl. Physiol..

[34] Herbert J.M., Bernat A., Maffrand J.P. (1992). Importance of platelets in experimental venous thrombosis in the rat. Blood.

[35] Fonseca R.J.C., Oliveira S.N.M.C.G., Melo F.R., Pereira M.G., Benevides N.M., Mourão P.A.S. (2008). Slight differences in sulfation of algal galactans account for differences in their anticoagulant and venous antithrombotic activities. Thromb. Haemost..

[36] Becker C.F., Guimarães J.A., Mourão P.A., Verli H. (2007). Conformation of sulfated galactan and sulfated fucan in aqueous solutions: Implications to their anticoagulant activities. J. Mol. Graph. Model..

