# Supplementary material for: Anticoagulant and Antithrombotic Properties of Three Structurally Correlated Sea Urchin Sulfated Glycans and Their Low-Molecular-Weight Derivatives
Source: Mar Drugs. 2018 Aug 30;16(9):304. doi: 10.3390/md16090304 (PMC6163371; doi:10.3390/md16090304)
Supplement: Supplementary file 1 [file marinedrugs-16-00304-s001.pdf]

marine drugs

Supplemental Material

Anticoagulant and Antithrombotic Properties of Three Structurally Correlated Sea Urchin Sulfated Glycans and Their Low-Molecular-Weight Derivatives

Ariana A. Vasconcelos [1,2], Isabela D. Sucupira [1,2], Alessandra L. Guedes [1,3], Ismael N. Queiroz [1,2], Flavia S. Frattani [3], Roberto J. Fonseca [2,4], and Vitor H. Pomin [1,2,5,*]

[1] Program of Glycobiology, Institute of Medical Biochemistry Leopoldo de Meis, Federal University of Rio de Janeiro, Rio de Janeiro, RJ 21941-590, Brazil;
[2] University Hospital Clementino Fraga Filho, Federal University of Rio de Janeiro, Rio de Janeiro, RJ 21941-913, Brazil;
[3] Department of Clinical Analyses and Toxicology, School of Pharmacy, Federal University of Rio de Janeiro, Rio de Janeiro, RJ 21941-599, Brazil;
[4] Undergraduate Program in Pharmacology, Institute of Biomedical Sciences, Federal University of Rio de Janeiro, Rio de Janeiro, RJ 21941-902, Brazil;
[5] Department of BioMolecular Sciences, Division of Pharmacognosy, and Research Institute of Pharmaceutical Sciences, School of Pharmacy, University of Mississippi, Oxford, MS 38677-1848, USA.
* Correspondence: vpomin@olemiss.edu; Tel.: +1-662-915-3114

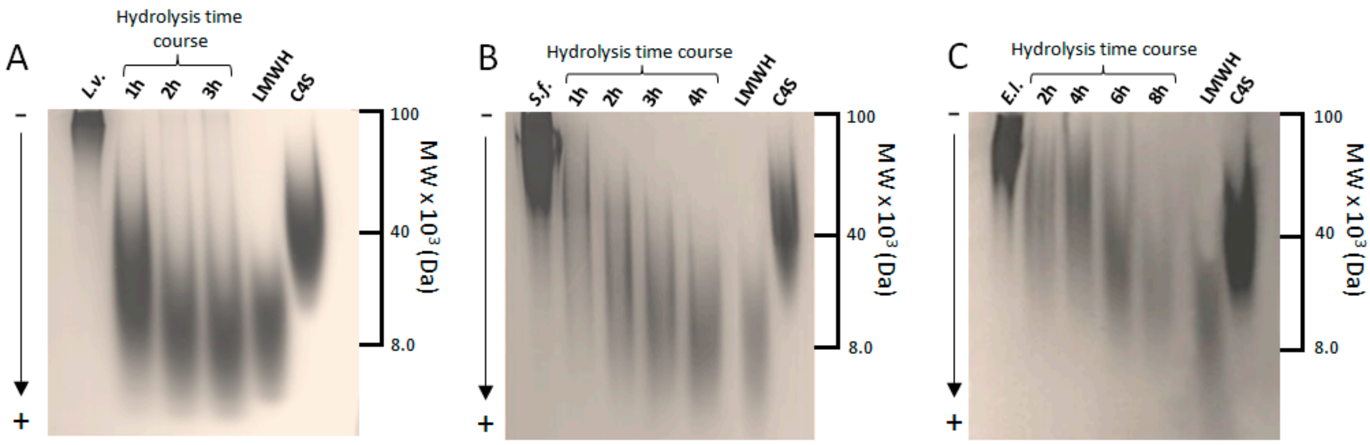

Figure S1. Molecular weight (MW) analysis by polyacrylamide electrophoresis (PAGE) of (**A**) native sulfated fucan from *Lytechinus variegatus* (*L.v.*) and derivatives obtained by acid hydrolysis (0.04 M HCl) within different time courses (1,2 and 3 h), (**B**) native sulfated fucan from *Strongylocentrotus franciscanus* (*S.f.*) and derivatives obtained by acid hydrolysis (0.4 M HCl) within different time courses (1, 2, 3 and 4 h), and (**C**) native sulfated galactan from *Echinometralucunter* (*E.l.*) and derivatives obtained by acid hydrolysis (1.0 M HCl) within different time courses (2, 4, 6 and 8 h). In both panels, MWs of marine sulfated glycans and low MW derivatives were estimated in comparison to two molecular markers: low-molecular weight heparin (LMWH) of 8 kDa and chondroitin 4-sulfate (C4S) of 40 kDa. The hydrolysis was performed as reported in Pomin et al. 2005; Queiroz et al. 2015; 2016 [1–4].

Table S1. ^{1}H and ^{13}C chemical shifts from sulfated glycans and derivatives studied in this work compared to values from references.

Glycan type / composing unit	Cross-peak	^{1}H This study	^{1}H Literature [5]	^{13}C This study	^{13}C Literature [5]
UFH / glucosamine	A1	5.38	5.40	96.4	98.6
	A2	3.26	3.28	57.4	60.0
	A3	3.67	3.68	69.3	ND
	A4	3.76	3.76	75.9	77.9
	A5	3.99	4.04	69.1	ND
	A6SO3	3.84	4.38	59.6	68.4
	A6 non SO3	4.32	4.27	66.0	68.4
UFH / iduronate	I1	5,20	5.21	98,6	101.3
	I2	4.33	4.34	75.5	80.0
	I3	4.18	4.20	68.9	71.3
	I4	4.09	4.10	75.7	78.0
	I5	4.79	4.81	69.3	71.4
**Glycan type / composing unit**	**Cross-peak**	**^{1}H This study**	**^{1}H Literature [6]**	**^{13}C This study**	**^{13}C Literature [6]**
LMWH / glucosamine	A1	5.39	5.42	96.4	99.5
	A2	ND	3.32	ND	60.0
	A3	3.38	3.68	72.7	ND
	A4	3.75	3.80	76.1	77.9
	A5	3.67	4.04	69.3	ND
	A6SO3	4.35	ND	65.7	ND
	A6 non SO3	3.85	ND	59.4	ND
LMWH / iduronate	I1	5.21	5.24	98.7	102.1
	I2	4.33	4.37	75.5	78.7
	I3	4.20	4.23	68.7	71.8
	I4	4.10	4.14	75.7	78.5
	I5	4.80	4.83	69.1	72.2
LMWH / nonreducing end 4,5-unsaturated uronic acid	ΔU1	5.49	5.53	96.8	100.1
	ΔU2	4.62	4.65	74.2	ND
	ΔU4	5.98	6.01	105.3	108.7
**Glycan type / composing unit**	**Cross-peak**	**^{1}H This study**	**^{1}H Literature [7]**	**^{13}C This study**	**^{13}C Literature [7]**
L.v. / 4-sulfated fucose	A1	4.90	5.10	96.2	98.5
	A2	3.65	3.86	64.6	68.8

	A3	3.83	4.02	73.8	76.6
	A4	4.54	4.72	77.3	79.9
	A5	4.28	4.45	64.1	67.0
	A6	1.05	1.25	13.3	15.8
**Glycan type / composing unit**	**Cross-peak**	**^{1}H This study**	**^{1}H Literature [7]**	**^{13}C This study**	**^{13}C Literature [7]**
*L.v.*hd / 4-sulfated fucose	A1	4.90	5.10	96.2	98.5
	A2	3.65	3.86	64.6	68.8
	A3	3.83	4.02	73.8	76.6
	A4	4.54	4.72	77.3	79.9
	A5	4.28	4.45	64.1	67.0
	A6	1.05	1.25	13.3	15.8
*L.v.*hd / desulfated fucose	B1	4.90	ND	96.2	ND
	B2	3.53	ND	66.0	ND
	B3	3.71	ND	73.1	ND
	B4	3.88	ND	66.3	ND
	B5	4.12	ND	64.1	ND
	B6	1.1	ND	17.72	ND
**Glycan type / composing unit**	**Cross-peak**	**^{1}H This study**	**^{1}H Literature [8]**	**^{13}C This study**	**^{13}C Literature [8]**
S.f. / 2-sulfated fucose	A1	5.33	5.33	96.7	96.8
	A2	4.53	4.55	74.5	75.5
	A3	4.08	4.10	75.7	75.9
	A4	4.06	4.07	70.9	71.2
	A5	4.41	4.42	68.3	68.6
	A6	1.22	1.25	16.97	17.3
**Glycan type / composing unit**	**Cross-peak**	**^{1}H This study**	**^{1}H Literature [4]**	**^{13}C This study**	**^{13}C Literature [4]**
*S.f.*hd / 2-sulfated fucose	A1	5.55	5.32	97.4	98.0
	A2	4.70	4.51	75.0	74.1
	A3	4.25	4.09	76.7	76.0
	A4	4.24	4.09	71.5	70.0
	A5	4.58	4.42	68.6	67.8
	A6	1.41	1.26	16.9	ND
*S.f.*hd / desulfated fucose	B1	5.22	5.06	98.5	96.0
	B2	4.24	4.11	67.7	70.1
	B3	4.12	3.94	77.0	76.2
	B4	4.09	3.94	67.9	67.8
	B5	4.44	4.23	68.7	67.8
	B6	1.32	ND	18.6	ND

Glycan type / composing unit	Cross-peak	^{1}H This study	^{1}H Literature [8]	^{13}C This study	^{13}C Literature [8]
E.l. / 2-sulfated galactose	A1	5.46	5.47	97.2	97.2
	A2	4.65	4.66	75.8	76.2
	A3	4.26	4.23	75.8	75.9
	A4	4.32	4.33	73.1	72.5
	A5	4.34	4.35	69.2	69.5
	A6	3.87	3.82	64.1	63.8
**Type**	**Cross-peak**	**^{1}H This study**	**^{1}H Literature [4]**	**^{13}C This study**	**^{13}C Literature [4]**
*E.l.*hd/ 2-sulfated galactose	A1	5.49	5.43	96.4	97.0
	A2	4.65	4.59	76.2	76.0
	A3	4.27	4.22	75.6	76.0
	A4	4.34	4.31	73.3	74.1
	A5	4.33	4.37	68.6	70.0
	A6	3.80	3.80	63.6	64.0
E.l. / desulfated galactose	B1	5.21	5.17	98.7	98.5
	B2	3.87	4.22	71.2	71.0
	B3	3.92	3.99	71.9	72.0
	B4	4.03	4.11	72.1	72.0
	B5	4.20	4.22	72.9	74.0
	B6	3.66	3.60	65.6	66.0

References

1. Pomin, V.H.; Pereira, M.S.; Valente, A.P.; Tollefsen, D.M.; Pavão, M.S.; Mourão, P.A.S. Selective cleavage and anticoagulant activity of a sulfated fucan: Stereospecific removal of a 2-sulfate ester from the polysaccharide by mild acid hydrolysis, preparation of oligosaccharides, and heparin cofactor II-dependent anticoagulant activity. *Glycobiology* **2005**, *15*, 369–681, doi:10.1093/glycob/cwi021.
2. Pomin, V.H.; Valente, A.P.; Pereira, M.S.; Mourão, P.A.S. Mild acid hydrolysis of sulfated fucans: A selective 2-desulfation reaction and an alternative approach for preparing tailored sulfated oligosaccharides. *Glycobiology* **2005**, *15*, 1376–1385, doi:10.1093/glycob/cwj030.
3. Queiroz, I.N.L.; Wang, X.; Glushka, J.N.; Santos, G.R.C.; Valente, A.P.; Prestegard, J.H.; Woods, R.J.; Mourão, P.A.S.; Pomin, V.H. Impact of sulfation pattern on the conformation and dynamics of sulfated fucan oligosaccharides as revealed by NMR and MD. *Glycobiology* **2015**, *25*, 535–547, doi:10.1093/glycob/cwu184.
4. Queiroz, I.N.L.; Vilela-Silva, A.C.E.S.; Pomin, V.H. Oligosaccharides from the 3-linked 2-sulfated alpha-L-fucan and alpha-L-galactan show similar conformations but different dynamics. *Glycobiology* **2016**, 26, 1257–1264, doi:10.1093/glycob/cww080.
5. Aquino, R.S.; Pereira, M.S.; Vairo, B.C.; Cinelli, L.P.; Santos, G.R.C.; Fonseca, R.J.C.; Mourão, P.A.S. Heparins from porcine and bovine intestinal mucosa: Are they similar drugs? *Thromb. Haemost.* **2010**, *103*, 1005–1015, doi:10.1160/TH09-11-0761.

6. Glauser, B.F.; Vairo, B.C.; Oliveira, S.N.M.C.G.; Cinelli, L.P.; Pereira, M.S.; Mourão, P.A.S. Structure and haemostatic effects of generic versions of enoxaparin available for clinical use in Brazil: Similarity to the original drug. *Thromb. Haemost*. **2012**, *107*, 302–314, doi:10.1160/TH11-09-0664.
7. Cinelli, L.P.; Castro, M.O.; Santos, L.L.; Garcia, C.R.; Vilela-Silva, A.C.E.S.; Mourão, P.A.S. Expression of two different sulfated fucans by females of *Lytechinus variegatus* may regulate the seasonal variation in the fertilization of the sea urchin, *Glycobiology* **2007**, *17*, 877–885, doi:10.1093/glycob/cwm058.
8. Pereira, M.S.; Vilela-Silva, A.C.E.S.; Valente, A.P.; Mourão, P.A.S. A 2-sulfated, 3-linked α-L-galactan is an anticoagulant polysaccharide. *Carbohydr. Res.* **2002**, *337*, 2231–2238.
